# Interaction of Passive Smoking and Diet Habits on Vitamin D Deficiency among Women of Reproductive Age in Rural Central China

**DOI:** 10.3390/nu15010126

**Published:** 2022-12-27

**Authors:** Yuan Zhang, Shiqi Lin, Jiajia Li, Xinming Song, Gong Chen, Lijun Pei

**Affiliations:** 1 Institute of Population Research/China Center on Population Health and Development, Peking University, Beijing 100871, China; 2 National Research Institute for Health and Family Planning, Beijing 100081, China

**Keywords:** women, nutrition, vitamin D, deficiency, passive smoking

## Abstract

Objective: Maternal dietary undernutrition is known to be associated with the risk of vitamin D (VD) deficiency. However, whether the risk of VD deficiency in women of reproductive age is influenced by the interaction between passive smoking and inadequate nutrition remains unknown. The aim of this study is to explore the interaction between passive smoking and dietary undernutrition on the risk of VD deficiency. Methods: A population-based case–control study including 1151 non-pregnant women of reproductive age between 18 and 40 years old was conducted in Henan Province, China from 2009 to 2010. Blood samples and information on exposure factors were collected. The prevalence of VD deficiency was estimated based on a result of serum 25-hydroxyvitamin D [25(OH)D] < 26.0 ng/mL. A multivariate logistic regression analysis was performed to explore the risk of VD deficiency. Results: The prevalence of VD deficiency was 61.5%. After adjusting for potential confounding factors, the interactions between passive smoking and no nutritional supplementation, passive smoking and insufficient egg intake, and passive smoking and insufficient milk dairy products intake were associated with the risk of VD deficiency, and the adjusted ORs were 3.40 (95% CI 2.26–5.13), 2.87 (95% CI 2.20–4.10), and 2.18 (95% CI 1.33–3.58), respectively. The interaction coefficients were calculated to be 2.35, 2.79, and 1.70, respectively, indicating there were significant interaction effects, as all of the coefficients were higher than 1. Conclusions: Our findings present that the risk of VD deficiency was potentially influenced by interactions between passive smoking and inadequate nutrition. Passive smoking might strengthen the effect of inadequate nutrition on the risk of VD deficiency among rural women of reproductive age. More attention should be paid to the health education and nutritional status improvement of women of reproductive age, especially in rural areas of developing countries.

## 1. Introduction

Vitamin D deficiency has become one of the most important public health issues in the world. Recent studies have shown a very high prevalence of VD deficiency among women of reproductive age. In developed countries, the prevalence of VD deficiency varies from 8% to 27% among pregnant women in the USA and Canada [1,2,3,4]. In Finland, 26% of women were observed to be VD deficiency in winter [5]. In developing countries, the rates of VD deficiency were about 32% among Brazilian women [6] and 35% among Bangladesh women [7] of reproductive age, and it was 42% among pregnant women in India [8]. Previous studies showed a high prevalence of around 40.7–75% of VD deficiency or insufficiency in different regions of China [9,10,11]. According to China’s National Nutrition and Health Survey (CNNHS) in 2010–2013, about 74.9% of Chinese pregnant women had VD deficiency. There were lots of other studies showing that VD deficiency continues to be a major public health problem worldwide [12,13].

Some risk factors relative to VD deficiency have been recognized, such as dietary habits and sun exposure [14,15]. Previous studies found that the Japanese elderly population with sufficient levels of 25(OH)D presented with a higher fish intake value (12 times per week) [16]. A cohort study indicated that the main dietary sources of VD among elderly Germans were fish, eggs, oils, bakery products, and milk or dairy products [17]. Higher nutritional supplementation, egg consumption, and fish consumption were associated with increased 25(OH)D concentrations in the mid-aged German population, while active smoking increased the risk of VD deficiency [18]. A lower level of VD in Mediterranean children was associated with passive smoke exposure [19].

The Mediterranean diet, which emphasizes the consumption of vegetables, fruits, whole grain cereals, and seafood, contrasts with the Western diet, which consumes large amount of sugar and high-fat dairy products, which are good sources of dietary vitamin D intake [20], while the traditional Chinese diet primarily consists of grains and vegetables, which incurs low VD levels.

VD deficiency has numerous adverse health outcomes for both women and their offspring [21]. VD deficiency among women of reproductive age is associated with an increased risk of adverse pregnancy outcomes, including intrauterine fetal growth retardation, impaired bone development, pre-eclampsia [22], prematurity, gestational diabetes mellitus [23], and a low birth weight [24], as well as long-term effects such as metabolic syndrome [25] and its complications [26], dyslipidemia, hyperuricemia [27], and cardiovascular diseases [28]. Therefore, it is crucial to prevent VD deficiency during their reproductive years.

Therefore, it is crucial to better understand the vitamin D status and identify the interactions between the risk factors for VD deficiency, especially in rural areas where women of reproductive age are at risk of having a suboptimal vitamin D status. Based on the aforementioned objectives, our hypothesis was that the risk of VD deficiency would be influenced by the interaction between passive smoking and inadequate nutrition among women of reproductive age.

## 2. Methods

### 2.1. Study Design and Population

As part of the Birth Defects Monitoring and Comprehensive Intervention Project, from December 2009 to February 2010, a population-based study was performed in Henan Province, China; this design has been published previously [29,30]. In brief, the project obtained a representative sample of married, non-pregnant women in Henan Province through multistage cluster sampling methods. From all of the 158 counties, four counties were chosen at random for the first stage. From each county, 10 towns were chosen at random for the second stage. From each town, five villages were chosen at random for the third stage. From each community, 10 women of reproductive age who were not pregnant were chosen at random, together with their spouses for the fourth stage. The inclusion and exclusion criteria were (a) married women with local hukou (household registration), (b) being of reproductive age (18–40 years old), (c) not being presently pregnant, (d) residents of the research countries, (e) being without any severe illness, such as those of the heart, liver, kidney, metabolic, blood, or other system diseases or malignancies. A total of 1151 women who intended to become pregnant were recruited between December 2009 and February 2010 as baseline participants.

### 2.2. Collection of Data and Blood Sample

Face-to-face interviews with the participants and their families were conducted by well-trained healthcare professionals to collect baseline information such as the demographic and socioeconomic characteristics of the women and their husbands: the women’s disease and treatment history, history of adverse pregnancy outcomes, history of family genetic diseases, history of drug use and treatment, general health status, physical examination, dietary habits (frequency of dietary and nutritional intake), environmental exposure factors, and behavioral factors. At the baseline, the health care providers also collected fasting venous blood samples (8 mL) for each woman participant. The time period of collection was from December 2009 to February 2010. The samples were prepared by centrifugation right after their collection, and then, they were stored at −80 °C at Peking University prior to analysis.

Before the on-site investigation, all of the staff participating in the investigation and biological sample collection were trained to ensure that all of the healthcare workers and health professionals involved in this investigation understand the methods of the investigation design and were very familiar with the contents of the questionnaire, and they clarified the investigation progress. The quality control personnel conducted spot checks and evaluations on the questionnaires and survey forms in the study area, and the qualified rate of sampling inspection was above 95%.

During the baseline survey, the study protocol was reviewed and approved by the Institutional Review Board of Peking University Health Science Center, and written informed consent was obtained from each participant prior to completing the questionnaire and drawing blood samples.

### 2.3. Measurement of Vitamin D

**Figure 1:**
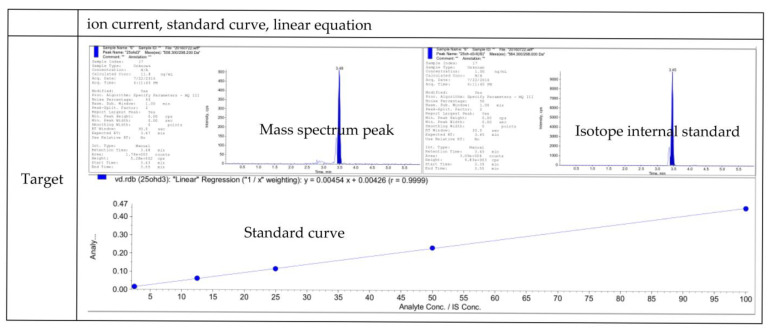
Quality control of the assessment of vitamin D status.

Serum 25-hydroxyvitamin D (25(OH)D) concentration was the parameter of choice for assessing the vitamin D status. The serum hydroxyl vitamin D3 concentrations of 1151 samples were quantitatively measured by high-performance liquid chromatography–tandem mass spectrometry (HPLC-MS/MS, Ultimate 3000-API 3200 Q TRAP). The quality control of the assessment method is shown in Figure 1. Additionally, the standardized tests were conducted to obtain the serum 25(OH)D data from all of the women during the physical examination. As the clinical normal reference range of serum vitamin D was 26.0–65.0 ng/mL, the serum vitamin D < 26.0 ng/mL was defined as vitamin D deficiency, and ≥26.0 ng/mL was defined as vitamin D sufficiency [31].

### 2.4. Definitions of Nutritional Supplementation and Passive Smoking

A woman was recognized as having taken nutritional supplementation if she regularly took any nutritional supplements, including vitamins, minerals, and their combined products within one month before the survey.

Passive smoking was defined as living with or working with tobacco smokers and being exposed to tobacco smoke for at least 15 min daily according to the WHO Guidelines for the Conduct of Tobacco Smoking Survey of the General Population [32].

In this study, almost none of the women smoked, which was consistent with the marked gender differences in terms of smoking prevalence, i.e., over half of Chinese adult men smoke, but few women smoke [33]. However, non-smoking women may still be subject to passive smoking.

### 2.5. Statistical Analysis

**Figure 2:**
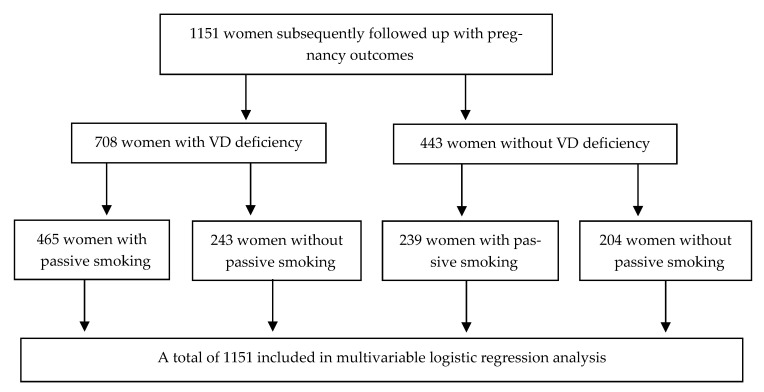
Participant flow chart of effort of passive smoking and inadequate dietary nutrition on the risk of vitamin D deficiency analysis.

The differences in exposure factors between vitamin D deficiency and sufficiency, including demographic characteristics, socioeconomic status, general health status, lifestyle and behaviors, and frequency of dietary intake, were examined by the Chi-square test in the univariate analysis. The multivariable logistic regression analysis using variables with significant differences in the univariate analysis was performed to assess the effects of the exposure factors on the risk of VD deficiency. The risk of VD deficiency was assessed by estimating the odds ratio (OR) and its 95% confidence interval (CI). Then, these values were selected for the interaction analysis. The participant flow chart was shown in Figure 2.

To estimate the interaction between passive smoking and nutritional supplementation on the risk of VD deficiency, the women of reproductive age were divided into four groups: women who did not experience passive smoking and used nutritional supplementation (reference group); those who did not experience passive smoking and did not use nutritional supplementation; those who used nutritional supplementation and experienced passive smoking; those experienced passive smoking and did not use nutrition supplementation. Likewise, in the analyses of the interaction between passive smoking and egg intake and between passive smoking and milk or dairy products intake, the women were also divided into four groups. In all of the three analyses, the women without an adverse exposure (i.e., those who did not experience passive smoking and used nutrition supplementation or related dietary intake) were considered as the reference group.

To evaluate the *E*_1_ × *E*_2_ (exposure factor 1 × exposure factor 2) interaction between the two factors, a multivariable logistic regression analysis was performed. An adjusted OR was calculated to simultaneously estimate the interaction effect between the two exposure factors after controlling for potential confounding variables. The coefficient of this interaction term then determined whether an interaction existed:

$$Y=\alpha +\beta_{e1}E_{1}+\beta_{e2}E_{2}+\beta_{e1e2}E_{1}*E_{2}$$

where *Y* was the log odds of disease, *E* was the exposure factor, and *E*_1_ ∗ *E*_2_ was the interaction term. The coefficients *β_e_*_1_, *β_e_*_2_, and *β_e_*_1*e*2_ were determined by regression analysis. The *β_e_* indicated the regression coefficient of exposure factor alone (passive smoking or no nutrition supplementation). *β_e_*_1*e*2_ was called the interaction coefficient. If *β_e_*_1*e*2_ was greater than 1, then the environmental factor (passive smoking) may strengthen the effect of nutritional factors (nutrition supplementation, egg intake, or milk or dairy products intake) on the risk of VD deficiency, indicating a significant interaction between the two exposure factors, while a *β_e_*_1*e*2_ value that is less than 1 indicates no significant interaction between the two exposure factors.

All of the data were analyzed using SPSS software (version 20.0; SPSS Inc., Chicago, IL, USA). Having a *p* value < 0.05 was considered to be a significant difference.

## 3. Results

### 3.1. Distribution of Vitamin D Deficiency among Women of Reproductive Age in Rural Central China

The distribution of vitamin D levels was positively skewed, and the median was 20.9 (95% CI 13.6–34.6) ng/mL among 1151 women of reproductive age in rural Central China. Additionally, the prevalence of VD deficiency was 61.5% according to the general clinical reference range.

Table 1 shows the distribution of demographic characteristics and socioeconomic status between the VD deficiency and VD sufficiency groups. The results showed there were significant differences in the women’s education and family annual income between the two groups (*p* < 0.001).

Table 2 shows the distribution of women’s exposure factors between the VD deficiency and VD sufficiency groups. There were significant differences in gravidity, passive smoking, nutritional supplementation, and the intake frequencies of eggs and milk or dairy products between the VD deficiency and VD sufficiency groups (*p* < 0.001 or 0.05).

### 3.2. The Risk of Vitamin D Deficiency by Multivariable Logistic Regression Analysis

The vitamin D level was taken as a dependent variable, and the aforementioned statistically significant variables were taken as independent variables to build the logistics regression model. The findings showed that regular nutritional supplement had protective effects on VD deficiency (OR 0.51, 95% CI 0.39–0.68). Compared with the family annual income CNY ≥ 10,000, the risks of VD deficiency for family annual income CNY 5000 and CNY < 5000 were 1.58 (95% CI 1.19–2.08) and 4.08 (95% CI 1.93–8.62), respectively. The women’s gravidity value being ≥ 2 and experiencing passive smoking were associated with an increased risk of VD deficiency (OR 1.85, 95% CI 1.33–2.57; OR 1.66, 95% CI: 1.28–2.24). The risk of VD deficiency for consuming eggs less than 3 times per week was 1.69 (95% CI 1.24–2.29) compared to that due to consuming eggs every day (Table 3).

### 3.3. Interaction between Passive Smoking and Inadequate Nutrition on the Risk of Vitamin D Deficiency

The interaction between passive smoking and inadequate nutrition on the risk of VD deficiency is presented in Table 4. Using women who did not experience passive smoking and used nutrition supplementation as the reference group (Model 1), the interaction between passive smoking and no nutritional supplementation was associated with an increased risk of VD deficiency (OR 3.40, 95% CI 2.26–5.13) after adjusting the confounding factors, including education, family annual income, and gravidity. The interaction coefficient was 2.35, which was higher than 1, showing a significant interaction between the two factors. Using women who did not experience passive smoking and had a sufficient egg intake level per week as the reference group (Model 2), the interaction between passive smoking and having an insufficient egg intake per week increased the risk of VD deficiency (OR 2.87, 95% CI 2.00–4.10) after adjusting the confounding factors. The interaction coefficient was 2.79, which was higher than 1, showing a significant interaction between the two factors. Using women who did not experience passive smoking and with sufficient levels of milk or dairy products intake as the reference group (Model 3), the interaction between passive smoking and insufficient milk or dairy products intake increased the risk of VD deficiency (OR 2.18, 95% CI 1.33–3.58) after adjusting the confounding factors. Again, the interaction coefficient was 1.70, which was higher than 1, showing a significant interaction between the two factors.

## 4. Discussion

### 4.1. Factors Influencing Vitamin D and Their Interaction

This study was the first epidemiological investigation to explore the potential effects of the interaction between passive smoking and inadequate nutrition on the risk of VD deficiency among rural Chinese women of reproductive age. Our findings demonstrated that passive smoking increased the risk of VD deficiency. China has implemented nationwide tobacco control policies since the 1980s. However, due to the influence of the social and cultural atmosphere, most women of reproductive age were unable to change the smoking habits of their husbands, family members, or other contacts, nor could they improve their rate of passive tobacco exposure in working and living environments, especially in rural areas where the economy is relatively underdeveloped. Previous studies showed that, when they were compared to women who had never smoked, women who currently smoked had a strikingly lower intake of vitamin D and calcium [34]. Research in the USA and Korea found that when they were compared to those who smoked actively, second-hand smoking adolescents were even more likely to be VD deficiency, and this is probably because active smoking often occurs outdoors and thus, it increases the smokers’ exposure to sunlight, while passive smoking occurs mostly indoors. The interaction between cigarette nicotine and calcium receptors may affect intestinal calcium absorption, and consequently, this will reduce the calcium levels [35].

Our findings indicated that nutritional supplementation, including with vitamins, minerals, and combined products, was one of the protective factors of VD deficiency, suggesting that food fortification should be encouraged. Vitamin D supplementation for women of reproductive age should be promoted, as there is evidence from the USA, where the vitamin D level in women’s body was found to correlate with the consumption of milk fortified with vitamin D, and from Norway, where cod liver oil is widely used to prevent VD deficiency. Although nutritional supplementation was one of the protective factors of VD deficiency in our study, the proportion of taking nutritional supplements among rural women of reproductive age was low: it is only at 26.5% in Central China. Moreover, the Chinese traditional diet structure is mainly composed of vegetables and grains, while foods rich in vitamin D are rarely consumed. According to a survey conducted by the Chinese Nutrition Society in recent years, the average dietary calcium intake of urban and rural residents in China is 412.4 mg/day and 321.4 mg/day, respectively, which is only about half of the daily calcium intake (800 mg/day) recommended by the Chinese Nutrition Society. Therefore, vitamin D levels are generally lower in the Chinese population.

Our previous research found that having a lower socioeconomic status is the risk factor for VD deficiency in women of reproductive age and having a maternal low socioeconomic status may even strengthen the risk of VD deficiency, resulting in spontaneous miscarriage [30]. The important findings in this study were that the interactions between passive smoking and not taking nutrition supplementation (OR 3.40, 95% CI 2.26–5.13), passive smoking and insufficiently consuming eggs (OR 2.87, 95% CI 2.20–4.10), and passive smoking and insufficiently consuming milk dairy products (OR 2.18, 95% CI 1.33–3.58) were all associated with an increased risk of VD deficiency. The present study adds new evidence of the interaction between passive smoking and nutrition, which have been reported by earlier research, i.e., sufficient nutrition could significantly reduce the risk of VD deficiency associated with passive smoking. In other words, passive smoking may strengthen the effect of inadequate nutrition on the risk of VD deficiency. All of the three studies suggested the importance of focusing on the vitamin D status of women at reproductive age.

### 4.2. Strengths and Limitations

A major strength of this study was that it is based on a women’s reproductive health study with a representative and large sample population, a good quality of control, and a higher measurement accuracy when it is compared to those involving enzyme-linked immunosorbent assays (ELISA) and chemiluminescent immunoassays (CLIA), which are the commonly used detection methods in rural areas of developing countries. The likelihood of recall bias was low because the information on primary exposures and blood samples was obtained simultaneously during the baseline survey. A potential limitation was that the time of the women’s exposure to sunlight was unavailable. Henan Province is located in Central China, with a medium latitude of around 35° N, four distinct seasons, and many sunshine hours in summer, while the winter is cold and hazy. The serum 25(OH)D levels may have been a little underestimated because the blood samples for this study were collected in the winter when the daylight and temperatures are lower than they are in the summer, people tend to have less outdoor activities, and the amount of vitamin D synthesized by the sun’s ultraviolet radiation on the skin is relatively low [36]. One other limitation is that passive smoke was measured by history alone, and there was no objective validation by use of biomarkers such as cotinine levels. However, the blood samples and the behavioral and dietary factors were collected simultaneously, so this possibly underestimated vitamin D level would not affect the evaluation of the association. Further studies should collect the subject’s blood sample in four seasons to better evaluate the potential interaction effect of dietary intake and other exposures on the risk of VD deficiency in a larger cohort population.

### 4.3. Suggestions Based on Research Findings

Our findings suggested that passive smoking was not only associated with the risk of VD deficiency, but its synergetic effects with inadequate nutrition might further increase the risk of VD deficiency. In our study, the rate of passive smoking among women was 61.2%. In addition, 97% of the participants in our study were rural women of reproductive age who may not only be expose to passive smoking, but they may also suffer from an inadequate nutritional intake. The combination of the two exposure factors may increase the risk of VD deficiency, especially for rural women of reproductive age, who are a special population, carrying a series of important tasks such as pregnancy and breastfeeding. VD deficiency not only affects maternal health, but it also affects the growth and development of fetuses and infants, given the known increased demand for vitamin D during pregnancy. Lots of studies have shown that low maternal vitamin D levels are associated with a variety of adverse pregnancy outcomes [37], including miscarriage, a baby that is small for its gestational age [38], and low-birth-weight infants [39]. Periconceptional VD deficiency has been found to be associated with the risk of infantile hypocalcemia, possible abnormal brain development, as well as infantile rickets. The roles of vitamin D playing in adverse health outcomes, during the perinatal and lactating periods, have raised considerable attention. Therefore, it suggested that women of reproductive age should avoid passive smoking and improve their nutrition status.

## 5. Conclusions

Vitamin D deficiency is still a common phenomenon among women of reproductive age in rural Central China. The risk of women’s VD deficiency may be potentially influenced by a lower daily intake of eggs and exposure to passive smoking. Additionally, the interaction between passive smoking and inadequate nutrition may increase the risk of VD deficiency. It suggested that the government and public should pay more attention to the health education and nutritional status improvement of women of reproductive age. Additionally, it was still necessary to implement smoking control strategies and strengthen the intervention of smoking cessation to protect women of reproductive age from tobacco exposure through joint efforts in many areas, especially in rural areas of developing countries.

## Figures and Tables

**Table 1:** Demographic characteristics and socioeconomic status between VD deficiency and VD sufficiency group.

Demographic Characteristics and Socioeconomic Status	VD Deficiency (*n* = 708)		VD Sufficiency (*n* = 443)		χ^2^	*p*
	*n*	%	*n*	%		
Age						
<25	214	30.23	145	32.73	2.641	0.267
25–	279	39.41	183	41.31		
30–	215	30.37	115	25.96		
Ethnicity						
Han	693	97.88	434	97.97	0.010	0.920
Minority	15	2.12	9	2.03		
Education						
Primary school or below	58	8.19	22	4.97	12.268	0.002
Junior middle school	482	68.08	278	62.75		
High school or above	168	23.73	142	32.13		
Occupation						
Enterprises	32	4.52	30	6.77	3.091	0.378
Business or service	64	9.04	39	8.80		
Agriculture or industry	99	13.98	55	12.42		
Housework	513	72.46	319	72.01		
Family annual income(Yuan) ^1^						
<5000	51	7.20	9	2.03	31.694	<0.001
5000–	260	36.72	120	27.09		
10,000–	395	55.95	314	70.88		

^1^ RMB CNY 5000 is roughly equivalent to USD 718 and CNY 1000 is roughly equivalent to USD 1436.

**Table 2:** Distribution of women’s exposure factors between VD deficiency and VD sufficiency group.

Exposure Factor	VD Deficiency		VD Sufficiency		χ^2^	*p*
	(*n* = 708)		(*n* = 443)			
	*n*	%	*n*	%		
BMI						
<18.5	42	5.93	34	7.67	2.629	0.453
18.5–	425	60.03	272	61.4		
24–	171	24.15	102	23.02		
28–	68	9.6	34	7.67		
Gravidity						
0	165	23.37	136	31.41	23.733	<0.001
1	258	36.54	191	44.11		
≥2	283	40.08	116	26.79		
History of chronic diseases ^1^						
Yes	33	4.66	18	4.06	0.235	0.628
No	674	95.2	425	95.94		
Passive smoking						
Yes	465	65.68	239	53.95	15.778	<0.001
No	243	34.32	204	46.05		
Alcohol consumption						
Yes	12	1.69	6	1.35	0.205	0.651
No	696	98.31	437	98.65		
Nutritional supplement						
Yes	149	21.05	155	34.99	27.258	<0.001
No	559	78.95	288	65.01		
Fresh meat intake						
≥4 times per week	77	10.89	59	13.32	3.370	0.185
1–3 times per week	307	43.42	204	46.05		
≤3 times per month	323	45.68	180	40.63		
Fish and shrimp intake						
≥1 time per week	48	6.79	31	7	4.572	0.102
1–3 times per month	84	11.88	72	16.25		
Rarely	575	81.33	340	76.75		
Egg intake						
Everyday	191	27.02	163	36.79	25.776	<0.001
4–6 times per week	158	22.35	123	27.77		
≤3 times per week	358	35.44	157	50.61		
Milk or dairy products intake						
≥4 times per week	113	15.98	94	21.22	6.309	0.043
≥1 time per month	226	32	120	21.09		
Almost never	368	52.05	229	51.69		
Beans and soy products intake						
Everyday	171	24.19	119	26.86	6.677	0.083
4–6 times per week	130	18.39	100	22.57		
1–3 times per week	188	26.59	114	25.73		
≤3 times per month	218	30.83	110	24.83		
Vegetables and fruits intake						
Everyday	548	77.51	346	78.1	1.063	0.588
4–6 times per week	100	14.14	67	15.12		
≤3 times per week	59	8.35	30	6.77		

^1^ Chronic diseases include hypertension, hyperlipidemia, heart disease, diabetes, thyroid disease, chronic kidney disease, systemic lupus erythematosus, and rheumatoid arthritis, etc.

**Table 3:** Multivariable logistics regression analysis of factors affecting VD deficiency.

Factor	β	S.E.	Wald	Sig	OR (95% CI)
**Education**					
High school or above			2.816	0.245	1.00
Junior middle school	0.444	0.291	2.327	0.127	1.60 (0.88–2.76)
Primary school or below	0.175	0.145	1.443	0.230	1.19 (0.90–1.58)
**Family annual income (Yuan)**					
<5000	1.406	0.382	13.567	<0.001	**4.08 (1.93–8.62)**
5000–	0.454	0.143	10.144	0.001	**1.58 (1.19–2.08)**
≥10,000			20.825	<0.001	1.00
**Gravidity**					
0			16.967	<0.001	1.00
1	0.070	0.158	0.200	0.655	1.07 (0.79–1.46)
≥2	0.613	0.168	13.261	<0.001	**1.85 (1.33–2.57)**
**Passive smoking**					
No					1.00
Yes	0.505	0.130	14.982	<0.001	**1.66 (1.28–2.14)**
**Nutritional supplementation**					
No					1.00
Yes	−0.670	0.145	21.234	<0.001	**0.51 (0.39–0.68)**
**Egg intake**					
Everyday			12.731	0.002	1.00
4–6 times per week	0.101	0.172	0.345	0.557	1.11 (0.79–1.55)
<3 times per week	0.522	0.156	11.171	0.001	**1.69 (1.24–2.29)**
**Milk or dairy products intake**					
≥4 times per week			3.298	0.192	1.00
≥1 time per month	0.140	0.197	0.505	0.477	1.15 (0.78–1.69)
Almost never	−0.138	0.180	0.581	0.446	0.87 (0.61–1.24)

The bold in the OR (95% CI) part indictes the Statistical significance.

**Table 4:** The interaction between passive smoking and nutrition on the risk of VD deficiency.

		VD Deficiency	VD Sufficiency	OR (95% CI)	Regression Coefficient	Interactive Coefficient
		(*n* = 708)	(*n* = 443)			
**Model 1**						
Passive smoking	Nutritional supplementation					
No	Yes	52	77	1.00		
No	No	191	127	2.20 (1.43–3.39) *	0.788	-
Yes	Yes	97	78	1.94 (1.20–3.12) *	0.661	-
Yes	No	368	161	3.40 (2.26–5.13) *	1.224	2.35
**Model 2**						
Passive smoking	Egg intake(≥4 times per week)					
No	Yes	115	134	1.00		
No	No	127	70	1.89 (1.28–2.81) *	0.638	-
Yes	Yes	234	152	1.81 (1.30–2.51) *	0.592	-
Yes	No	231	87	2.87 (2.00–4.10) *	1.052	2.79
**Model 3**						
Passive smoking	Milk or dairy products intake (≥4 times per week)					
No	Yes	34	42	1.00		
No	No	208	162	1.32(0.79–2.20)	0.278	-
Yes	Yes	79	52	1.83 (1.02–3.28) *	0.606	-
Yes	No	386	187	2.18 (1.33–3.58) *	0.781	1.70

Models 1, 2, and 3, which were adjusted for education, family annual income, and gravidity. * *p* < 0.05. The bold in the OR (95% CI) part indictes the Statistical significance.

## Data Availability

The data presented in this study are available on request from the corresponding author.
